# Social Brain Energetics: Ergonomic Efficiency, Neurometabolic Scaling, and Metabolic Polyphenism in Ants

**DOI:** 10.1093/icb/icac048

**Published:** 2022-05-25

**Authors:** Zach N Coto, James F A Traniello

**Affiliations:** Department of Biology, Boston University, Boston, MA 02215, USA; Department of Biology, Boston University, Boston, MA 02215, USA; Graduate Program in Neuroscience, Boston University, Boston, MA 02215, USA

## Abstract

Metabolism, a metric of the energy cost of behavior, plays a significant role in social evolution. Body size and metabolic scaling are coupled, and a socioecological pattern of increased body size is associated with dietary change and the formation of larger and more complex groups. These consequences of the adaptive radiation of animal societies beg questions concerning energy expenses, a substantial portion of which may involve the metabolic rates of brains that process social information. Brain size scales with body size, but little is understood about brain metabolic scaling. Social insects such as ants show wide variation in worker body size and morphology that correlates with brain size, structure, and worker task performance, which is dependent on sensory inputs and information-processing ability to generate behavior. Elevated production and maintenance costs in workers may impose energetic constraints on body size and brain size that are reflected in patterns of metabolic scaling. Models of brain evolution do not clearly predict patterns of brain metabolic scaling, nor do they specify its relationship to task performance and worker ergonomic efficiency, two key elements of social evolution in ants. Brain metabolic rate is rarely recorded and, therefore, the conditions under which brain metabolism influences the evolution of brain size are unclear. We propose that studies of morphological evolution, colony social organization, and worker ergonomic efficiency should be integrated with analyses of species-specific patterns of brain metabolic scaling to advance our understanding of brain evolution in ants.

## Introduction

Sociality is considered a major evolutionary transition in the history of life (Szathmáry and Smith 1995) and group living increases biological complexity across diverse clades. In association with changes in habitat, diet, and body size, social evolution may be an important selective force of metabolic scaling—the relationship between size and metabolism. In ungulates, for example, an abundant, low-quality, grass-based diet likely favored increased body size, group size, and adaptive social structure (Jarman 1974; Szemán et al. 2021). Social evolution in primates reflects similar patterns of habitat- and diet-related selection for increased body size, group size, and social complexity (Clutton-Brock and Harvey 1977; Swedell and Plummer 2019) that coevolve with metabolic rate (Pontzer et al. 2009; Kozlowski et al. 2020). Although metabolic scaling is well-documented in mammals (White and Kearney 2014), the influences of social evolution on metabolism are not well-understood.

**Fig. 1:**
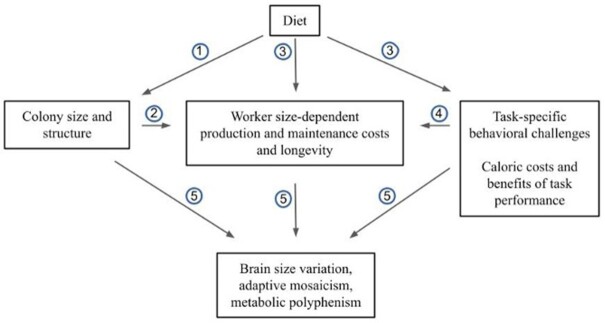
Evolutionary shifts in diet and collaterally foraging ecology impact colony social organization, worker behavioral demands, and energy availability, which ultimately drive brain evolution and impact colony fitness (Sayol et al. 2020; Azorsa et al. 2022). (1) Colony size is limited by energy availability through the biomass of food providing caloric input for colony operations required for growth and reproduction. (2) Colony size may be associated with worker production and maintenance costs and longevity, and potentially the evolution of polymorphism and adaptive worker size distributions. Polymorphic workers may have size-dependent and species-specific metabolic requirements for production, growth, maintenance, and task performance. (3) Worker metabolic requirements may be met through diet, such as variation in carbohydrate, protein, and lipid content. (4) High production and growth costs of relatively large workers (majors) may be offset by their low maintenance costs (Shik 2010) and contributions to defense that reduce the mortality of workers of other size classes. (5) Increased colony size, and associated reliance on collective behavior and division of labor, may select for reduced worker brain investment or adaptive mosaic structure influenced by task-specific behavioral demands. The relationship between task performance, brain size and structure, body size and structure, and metabolic cost, and the impacts of collective behavior and division of labor on task efficiency determine colony ergonomics. In association with variation in brain size, worker size-specific behavioral demands may influence brain metabolic polyphenism in polymorphic species. Polymorphic workers may exhibit differences in brain metabolic demands relative to size equivalent monomorphic workers due to behavioral demands associated with polymorphism, and this may be reflected in variation in metabolic pathways across size classes.

Social evolution may influence body size and metabolic scaling in insects (Coto and Traniello 2021). Queens, for example, may be several orders of magnitude larger than workers. Queens, workers, and males also vary significantly in longevity (Kramer and Schaible 2013) and worker longevity is associated with reduced mass-specific worker metabolic rates (Giraldo et al. 2021). Metabolic tradeoffs may be minimized by distributing reproductive and non-reproductive physiologies in different-size bodies (Friedman et al. 2020). Additionally, sterile workers may show strong body-size variation, functionally specialized adaptive morphologies, and at the colony level demographic distributions that contribute to division of labor and efficient task performance (Oster and Wilson 1978; Yang et al. 2004; Wills et al. 2018). Therefore, there may be significant differences in metabolic scaling among polymorphic workers due to divergent worker functions. Worker whole-body metabolic rate typically scales hypometrically with body mass (Chown et al. 2007; Riveros and Enquist 2011): larger workers have lower mass-specific metabolic rates than smaller workers. Worker metabolic rate may also scale hypometrically with colony size; average worker metabolic rate is lower in larger colonies in some species (Waters et al. 2017), but not others (Mason et al. 2015). Mechanisms underlying colony-level metabolic scaling may be clade-specific (Waters and Harrison 2012) and hypometric scaling may depend on costs associated with collective behavior (Ko et al. 2022) as well as the demography of major (defensive) workers with reduced mass-specific metabolic rates (Shik 2010). Sociality, among other factors, may impose behavioral and/or cognitive challenges favoring the evolution of brain size and adaptive mosaic structure (Dunbar and Shultz 2017, 2021; DeCasien and Higham 2019), but how sociality influences patterns of brain and body metabolic scaling is not well-understood. Here, we integrate theories of brain evolution with concepts of social insect colony organization to examine the impacts of social evolution on brain size and metabolism in ants. In Fig. 1, we describe conceptual relationships between diet, social complexity, colony ergonomics, worker longevity, and adaptive brain size, structure, and metabolic cost.

### Expensive brains, brain metabolic scaling, and the metabolic costs of brain miniaturization in ants

The expensive brain hypothesis assumes disproportionately high brain metabolic costs relative to brain size. Neurometabolic expenses concern costs of ion gradient maintenance to generate action potentials (Attwell and Laughlin 2001; Yu et al. 2017) and synaptic processing (Harris et al. 2012; Pulido and Ryan 2021). Larger brains may therefore increase brain metabolic costs. This may require increase basal metabolic rate (Pontzer et al. 2016) and reduce investment in other organs (Aiello and Wheeler 1995; Isler and van Schaik 2009) or favor molecular mechanisms that increase energetic efficiency (Kamhi et al. 2016).

Few studies directly measure brain metabolism to test these theories. The frequently cited notion that the brain is an expensive organ (Aiello and Wheeler 1995) is derived from studies of humans and mice (Sokoloff 1960), but the idea that brain costs are disproportionately large in animal energy budgets is not generally supported (Mink et al. 1981; Kern 1985). Human brains, moreover, may not in fact be exceptionally costly (Herculano-Houzel 2012). Furthermore, the expensive brain hypothesis does not account for hypometric brain metabolic scaling. The degree to which brain metabolic cost constrains brain evolution may be dependent on how brain metabolism scales with brain size. Without such data, it is difficult to identify metabolic costs associated with variation in brain size and structure and predict their influences on brain evolution.

Minute insects, like other small animals, follow Haller's rule (Lüps 2010): they have greater relative brain size than larger-bodied species (Eberhard and Wcislo 2011; Polilov and Makarova 2017). Ants and other hymenopterans may exhibit diphasic brain size/body size scaling that show significant slope changes at a given body size (Seid et al. 2011) or a shift to isometric scaling as found in some protist-sized wasps (Groothuis and Smid 2017). High metabolic cost of neural tissue may constrain increasing relative brain size in small-bodied species (Seid et al. 2011), but this remains to be tested. Alternatively, low behavioral demands could relax selection for large relative brain size below a critical body size.

In any case, the relationship between brain size and behavior is actively debated (Chittka and Niven 2009) and it is unclear whether small-bodied species compromise behavioral performance. In social insects, the relationship between brain size *per se* and worker behavior is unclear (Godfrey and Gronenberg 2019) and the metabolic costs of brains that meet demands associated with individual or colony-level behavior are largely unknown. In ants, larger brain size may be associated with large colony size (Wehner et al. 2007). Brain size and mosaic structure may also depend on task-specific behavioral demands that correlate with worker size (Muratore et al. 2022), and are likely influenced by both diet and social complexity (Azorsa and Traniello 2022). In vespid wasps, the degree of sociality is negatively correlated with relative size of the mushroom bodies (centers of higher-order processing) possibly due to relaxed selection for worker brain investment in large colonies (O'Donnell et al. 2019). In bees, the degree of diet specialization, rather than sociality, may explain variation in mushroom body investment (Sayol et al. 2020). Moreover, mushroom body elaboration in the hymenoptera is associated with parasitoidism (Farris and Schulmeister 2010). The extent to which variation in brain size, structure, and metabolic cost is associated with worker behavioral repertoire and/or worker roles in emergent colony-level processes and division of labor may depend on how task demands are most efficiently met by individual and/or collective actions. Brain and body metabolic scaling analyses in combination with data on worker behavioral demands and colony ergonomics will be necessary to test hypotheses involving constraints and selection pressures involved in brain evolution.

### Colony ergonomics: worker production and maintenance costs in relation to colony-level efficiency

The size and demographic structure of an insect society and the task specializations of its workers are hypothesized to represent ecological adaptations to optimize colony reproductive success (Oster and Wilson 1978). This “factory within a fortress” concept of colony organization is based on colony *ergonomics*, defined as “the quantitative study of the distribution of work, performance, and efficiency in insect societies” (Wilson 1968). Colony fitness depends on energy, the “basic currency” of a colony and its operations. The labor contribution of an individual of a given worker physical caste and age is hypothesized to optimize fitness gains by maximizing the energetic (caloric) benefits and minimizing energetic costs of task performance (Wilson 1980) and otherwise improving colony productivity.

Relatively large workers (majors or “soldiers”) in ants, for example, may improve colony energy intake and nutrition by defense of food sources, transport of large food items, carbohydrate and lipid storage, and food processing. Majors may also lower worker losses during encounters with competitors or predators. The relatively high production costs of majors may be offset by low mass-specific metabolic rates that reduce their maintenance costs (Shik 2010). Alternatively, investment in large numbers of smaller minor workers may be sufficient to locate, defend, and successfully exploit food sources depending on resource distribution and competition. Selection for increased worker longevity may correlate with body size and depend on relative production and maintenance costs as well as mortality risk. If worker production costs are relatively higher than worker maintenance costs, selection may favor increased worker longevity. However, increased risk of mortality may select for reduced longevity (Giraldo et al. 2021). The evolution of major workers as defensive specialists may reduce the risk of extrinsic mortality and relax such selection.

Collective behavior may improve colony-level efficiency by reducing per-capita costs of task performance and enabling colonies to complete tasks beyond the ability of individual workers while reducing worker information-processing demands (Reséndiz-Benhumea et al. 2021). Collective or distributed intelligence requires worker behavioral and/or morphological adaptations, which in turn entail metabolic costs. The colony-level fitness benefits of collective behavior are known (Sasaki and Pratt 2018), but how these benefits relate to increases in net reduction in *per capita* energy use and, therefore, task efficiency is not always clear. For example, comparisons of nest site quality during colony emigration improve decision-making (Robinson et al. 2014), but the impact on net energy expenditure per worker is unknown. These impacts may include metabolic costs of information processing during nest assessment and quorum sensing, energetic benefits to the colony accrued from time foraging that would otherwise be expended on nest searching, and reduced worker mortality and enhanced development of immatures derived from residing in an optimal nest site.

Maximizing a colony's energetic benefits and minimizing worker energetic costs likely depends on the net energetic benefit of each worker's task performance, and whether this is impacted by collective behavior and division of labor and/or by worker longevity. Ultimately, this is determined by worker body size, morphology, and physiology. Testing hypotheses regarding the impact of collective behavior and longevity on worker metabolism requires measurement of production and physiological maintenance costs. Worker brain evolution may impact colony ergonomics depending on how brain size, structure, and metabolic cost relate to worker task performance.

### Division of labor and behavioral demands in relation to brain size and metabolic scaling

Worker size, morphology, colony demography, and behavior evolve as systems of division of labor. Worker brain size and structure are predicted to coevolve with task performance that may vary in requirements for sensory processing, motor coordination, learning, and memory. Workers with diverse task repertoires appear to have proportionally larger mushroom bodies than task-specialists, which may show disproportional investment in brain compartments involved in peripheral-input or higher-order processing. In ant species characterized by strong worker size variation such as *Pheidole dentata* (Muscedere and Traniello 2012), *Pheidole rhea* (Gordon et al. 2017), and *Cephalotes varians* (Gordon et al. 2019), task-generalist minor workers have greater proportional investment in mushroom bodies than majors, which typically specialize on colony security or food processing. In the strikingly polymorphic leafcutter ant *Atta cephalotes*, proportional investment in the optic lobes is associated with task performance and worksite variation in ambient light conditions (Arganda et al. 2020). In respect to information-processing requirements for task performance in this species, mushroom bodies and antennal lobes are allometrically large in media workers (leaf harvesting) and the central complex is allometrically large in minim workers (fungus gardening and nursing; Muratore et al. 2022).

The impact of sociality and its consequences for worker morphological and behavioral adaptation, information-processing demands, and brain evolution requires further analysis. Large colony size and/or worker polymorphism may involve behavioral demands requiring increased brain size or brain compartment size for processing qualitative and quantitative social information as well as task-related cues and signals. However, social life and collective intelligence may adaptively increase *or reduce* brain size (DeSilva et al. 2021). Comparative analyses of brain size, proportional mushroom body, and antennal lobe investment across species varying in colony size and the nature of collective action have found positive (Wehner et al. 2007; Kamhi et al. 2016; Pahlke et al. 2021) as well as negative (Ehmer and Hoy 2000; Mares et al. 2005; O'Donnell et al. 2019) correlations among total brain size, brain compartment size, and colony size. The evolution of brain size and brain compartment size appear to be associated with colony size in monomorphic fungus-growing ants (Riveros et al. 2012). In the plant mutual *Pseudomyrmex spinicola*, which has monomorphic workers, mushroom body volume is positively correlated with colony size in foragers but negatively correlated in defensive specialists (Amador-Vargas et al. 2015), suggesting that task specialization rather than colony size *per se* is associated with behavioral demands that influence worker brain evolution.

Collective behavior and/or worker size-specific task repertoire may influence brain metabolism. Brain metabolic costs may differ substantially among workers that perform tasks independently or cooperatively. Furthermore, the cost of building and maintaining a brain that processes task-specific information may differ from that of a brain that processes and integrates information from diverse sensory modalities underpinning a broad task repertoire (Muratore et al. 2022).

Brain metabolic scaling data is necessary to determine how task diversity is associated with brain size, brain compartment size, and metabolic cost. If the evolution of increased brain size is constrained by brain metabolic costs that correlate with increased behavioral demands, then brain metabolic rate is expected to compose a high proportion of body metabolic rate and scale isometrically with respect to brain size and body metabolic rate. Brain metabolic cost would depend on behavioral demands independent of worker body size. However, brain metabolism may not be a high proportion of body metabolic rate and brain metabolism may scale hypometrically with brain size and body metabolic rate. Hypometric scaling implies that increasing brain size may not consistently impose substantial metabolic costs, as the cost incurred by increasing brain size at small body size would be higher than the cost incurred at large body size. Brain metabolic scaling data can, therefore, be used to test hypotheses of whether metabolic costs constrain the evolution of brain size under selection associated with behavioral demands.

### Brain metabolic polyphenism in ants: brain metabolic costs, neuron density, and metabolic pathways

Ants may exhibit metabolic polyphenism—variation in metabolic pathways and metabolic costs associated with variation in body size and task-dependent behavioral demands. Major workers, for example, entail high production costs that may be offset by low mass-specific body metabolic rate (Shik 2010). In species in which majors also have larger absolute brain size (Muscedere and Traniello 2012; Muratore et al. 2022), we expect this subcaste will exhibit lower mass-specific brain metabolic rate. Additionally, task-specialists have relatively smaller mushroom bodies than task-generalists (Muscedere and Traniello 2012; Gordon et al. 2017, 2019; Muratore et al. 2022). Therefore, task-specialists likely have fewer behavioral demands (Muratore et al. 2022) and lower brain metabolic rates than task-generalists of the same body size if specialization reduces information processing and motor requirements. Complex division of labor and/or collective behavior may also reduce worker information-processing demands and lower brain size and operation and maintenance costs. Alternatively, if specialized tasks incur costs associated with defensive aggression by majors or if collective behavior lowers the cost of individual contributions to group actions, then task specialization and emergent colony-level behavior may select for adaptive variation in brain metabolic rates and the molecular pathways that provide energy.

**Fig. 2:**
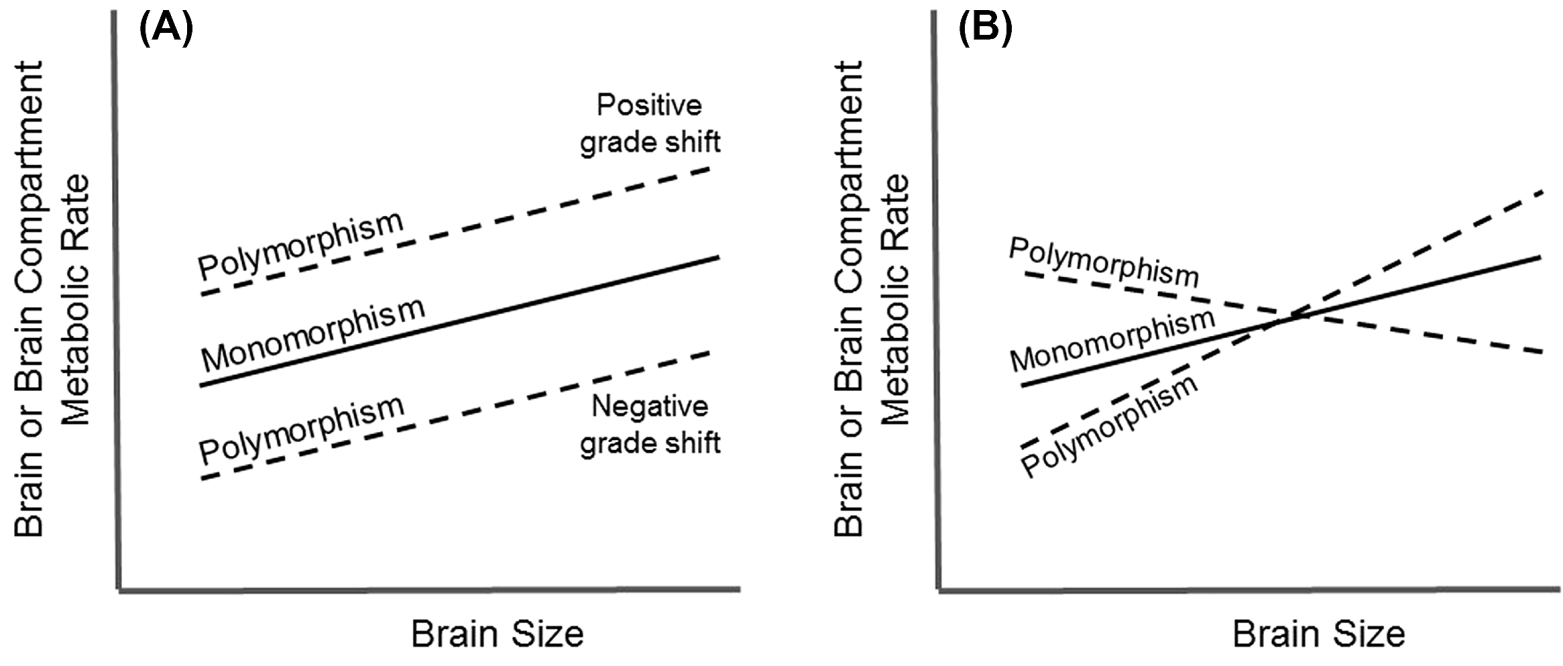
Theoretical associations of brain or brain compartment metabolic scaling in monomorphic or polymorphic species. Polymorphism may be associated with grade shifts **(A)** or changes in slope **(B)** in neurometabolic scaling relative to monomorphic species

Information-processing demands and associated brain metabolic costs may differ among morphologically differentiated workers, resulting in variation in brain metabolic scaling patterns between monomorphic or polymorphic species due to adaptive variation in brain metabolic rate associated with body size and correlated behavioral demands. Polymorphic workers may exhibit proportionally higher or lower brain metabolic costs than monomorphic workers, evident in positive or negative grade shifts (significant differences in y-intercepts) in brain metabolic scaling (Fig. 2A), and brain metabolic polyphenisms. Increased brain production costs of large polymorphic workers may be offset by decreased brain operation costs (Kamhi et al. 2016), resulting in a negative-scaling slope unique to polymorphic workers. Alternatively, monomorphic and polymorphic species may exhibit hypometric brain metabolic scaling. However, size reduction in polymorphic workers may not result in as disproportional an increase in brain metabolic demands as in monomorphic workers, reflected in an increase in the slope for polymorphic workers relative to monomorphic workers. Differences in slopes and y-intercepts of brain metabolic scaling may result in significant differences in brain metabolic rate between monomorphic and polymorphic workers (Fig. 2B).

Variables other than brain size may reflect behavioral demands (Godfrey and Gronenberg 2019) and impact brain metabolic costs. Brain cell number and density exhibit significant interspecific variation across ants (Godfrey et al. 2021) and the density of microglomeruli, synaptic complexes in the mushroom body, differ between subcastes in some (Gordon and Traniello 2018; Gordon et al. 2019), but not all (Groh et al. 2014) polymorphic species. Brain metabolic cost may be directly proportional to neuron number (Herculano-Houzel 2012) and driven by synaptic processing (Harris et al. 2012; Pulido and Ryan 2021). Additionally, neuron size and axon wiring volume strongly impact neuronal metabolic costs (Niven 2016). Therefore, variation in the identity, size, and density of neurons such as mushroom body Kenyon cells and their synaptic processing demands may result in variation in brain metabolic rate independent of brain volume. Such metabolic variation may occur in monomorphic or polymorphic species in association with behavioral variation (Amador-Vargas et al.2015).

Metabolic pathways that provide energy for task performance among polymorphic workers may vary depending on behavioral demands and work environment. For example, workers that perform tasks in subterranean low-oxygen/high CO_2_ environments may utilize glycolytic ATP production rather than aerobic respiration, or terminal electron acceptors such as fumarate as alternatives to oxygen (Spinelli et al. 2021). Shifts from aerobic respiration to aerobic glycolysis has also been shown to cause aggression in honey bees and other insects (Li-Byarlay et al. 2014; Rittschof et al. 2018). Therefore, ant workers specialized on defense may have higher glycolytic capacity and rapidly shift to aerobic glycolysis to fuel agonistic behavior. Additionally, while carbohydrates serve as the predominant brain energy substrate, other substrates such as fatty acids may be used (Rittschof and Schirmeier 2018) and be specific to worker size-classes and/or task specializations. Major workers can specialize on lipid storage (Kalife and Peeters 2021), and therefore, exhibit increased activity of fatty acid beta-oxidation pathways relative to minors. Brain metabolic pathways may be associated with the differential expression of metabolic genes, for example in defense-specialist majors of *A. cephalotes* (Muratore et al., submitted for publication). Polymorphism and division of labor may, therefore, be reflected in polyphenic metabolic pathways. Records of brain metabolic cost, analyses of the details of metabolic pathways, and assessments of molecular variation among workers that differ in task specialization are necessary to test this hypothesis.

### Conclusions and future research

A relationship between sociality and metabolism can be hypothesized from the correlated evolution of body size and group size in some social species, but patterns of metabolic scaling and collateral metabolic adaptations involved in social life are not well-understood. In social insects, the energetic benefits and costs of worker task performance are key to colony ergonomics and fitness, and metabolism is expected to be significant at the levels of the individual, cooperative group, and society. Ants provide an extraordinarily diverse array of societies in which body size and colony size may vary by four and six orders of magnitude, respectively, and the degree of social complexity, which concerns colony size, worker size-related task specialization, and collective intelligence, may influence the metabolic costs of behavioral performance. Energy use by the brain, which has generally been described as a significant expense, is likely to be an important cost component of overall metabolism, but this assumption needs to be tested. Worker brain size and mosaicism appear to reflect task-specific behavioral demands influenced by social complexity, but the impact of distributed cognition and group decision-making on brain metabolism require further analysis. The behavioral demands of sociality may favor species-specific patterns of brain metabolism. In polymorphic species, these demands may have selected for worker size- and task-related metabolic polyphenisms reflected in variation in brain metabolic scaling and differentiation of metabolic pathways to support behavior. Analyses of brain metabolic scaling are necessary to evaluate operational costs of the brain and allometries among its functionally specialized compartments that process sensory information and generate motor outputs controlling task performance. These studies will contribute to our understanding of worker size-dependent and species-specific metabolic constraints on brain evolution and, by extension, constraints on worker task efficiency and colony ergonomics.

## Notes

From the symposium “Causal mechanisms of interspecific metabolic scaling patterns’’ presented at the annual meeting of the Society for Integrative and Comparative Biology virtual annual meeting, January 3–February 28, 2022.
